# Delivering an in-Home Exercise Program via Telerehabilitation: A Pilot Study of Lung Transplant Go (LTGO)

**DOI:** 10.5195/ijt.2016.6201

**Published:** 2016-12-15

**Authors:** JIYEON CHOI, ANDREA L. HERGENROEDER, LORA BURKE, ANNETTE DEVITO DABBS, MATTHEW MORRELL, ANDI SAPTONO, BAMBANG PARMANTO

**Affiliations:** 1 DEPARTMENT OF ACUTE & TERTIARY CARE, SCHOOL OF NURSING, UNIVERSITY OF PITTSBURGH, PITTSBURGH, PENNSYLVANIA, USA; 2 DEPARTMENT OF PHYSICAL THERAPY, SCHOOL OF HEALTH AND REHABILITATION SCIENCE, UNIVERSITY OF PITTSBURGH, PITTSBURGH, PENNSYLVANIA, USA; 3 DEPARTMENT OF HEALTH & COMMUNITY SYSTEMS, SCHOOL OF NURSING, UNIVERSITY OF PITTSBURGH, PITTSBURGH, PENNSYLVANIA, USA; 4 DIVISION OF PULMONARY ALLERGY AND CRITICAL CARE MEDICINE, DEPARTMENT OF MEDICINE, UNIVERSITY OF PITTSBURGH SCHOOL OF MEDICINE & SCHOOL OF NURSING, UNIVERSITY OF PITTSBURGH, PITTSBURGH, PENNSYLVANIA, USA; 5 DEPARTMENT OF HEALTH INFORMATION MANAGEMENT, SCHOOL OF HEALTH AND REHABILITATION SCIENCE, UNIVERSITY OF PITTSBURGH, PITTSBURGH, PENNSYLVANIA, USA

**Keywords:** Home exercise program, Lung transplantation, Pulmonary rehabilitation, Telerehabilitation

## Abstract

We evaluated the feasibility, safety, system usability, and intervention acceptability of Lung Transplant Go (LTGO), an 8-week in-home exercise intervention for lung transplant recipients using a telerehabilitation platform, and described changes in physical function and physical activity from baseline to post-intervention. The intervention was delivered to lung transplant recipients in their home via the Versatile and Integrated System for TeleRehabilitation (VISYTER). The intervention focused on aerobic and strengthening exercises tailored to baseline physical function. Participants improved walk distance (6-minute walk distance), balance (Berg Balance Scale), lower body strength (30-second chair stand test) and steps walked (*SenseWear Armband®*). No adverse events were reported. Participants rated the program highly positively in regard to the technology and intervention. The telerehabilitation exercise program was feasible, safe, and acceptable. Our findings provide preliminary support for the LTGO intervention to improve physical function and promote physical activity in lung transplant recipients.

Due to notable progress in organ preservation, surgical techniques, and immunosuppression, the 5-year survival rate for lung transplant recipients has improved and currently is 53% (Yusen et al., 2014). Despite this success, several challenges remain. Due to deconditioning during the pre-transplant period, significant skeletal muscle weakness and reduced exercise capacity exist after transplantation (Bartels et al., 2011; Maury et al., 2008; Walsh et al., 2013). These factors pose barriers to achieving quality of life benefits after lung transplantation (Mathur, Levy, & Reid, 2008; Mathur, Reid, & Levy, 2004). Therefore, exercise has been recommended as a standard of care for lung transplant recipients (Rochester, Fairburn, & Crouch, 2014).

Despite this recommendation, prior studies document that while lung function improves after transplant, limitations in physical function continue. Lung transplant recipients have diminished exercise capacity (40–60% of predicted) as long as two years following transplant (Walsh et al., 2013), walk fewer steps and spend less time doing moderate to intense activity than age-matched healthy adults (Langer et al., 2009). These findings are particularly concerning because of the extensive resources that are expended during the process of transplant candidate evaluation, surgery, and recovery.

Pulmonary rehabilitation programs are designed to increase exercise tolerance and are therefore, widely used for patients with chronic respiratory disease, including lung transplant recipients (Spruit et al., 2013). However, despite demonstrated benefits in improving muscle strength, endurance and health-related quality of life, pulmonary rehabilitation programs are underutilized, with up to half of referred patients never attending or failing to complete the program (Rochester et al., 2015). This outcome has been attributed to a variety of factors, including travel and transportation issues, lack of support from family members, and perception of minimal benefit from participants (Jones et al., 2014; Keating et al., 2011).

Telerehabilitation offers an alternative approach that may better meet patient needs. While no previous studies have tested home exercise using telerehabilitation in lung transplant recipients, several studies have evaluated its benefits in patients with moderate to severe chronic obstructive pulmonary disease (COPD) (Holland et al., 2013; Marquis, Larivee, Saey, Dubois, & Tousignant, 2015; Tousignant et al., 2012; Zanaboni, Lien, Hjalmarsen, & Wootton, 2013). Findings revealed improvements in physical function. No adverse events were reported. Participants were able to master use of the technology with minimal difficulty.

Lung transplant recipients face challenges that make telerehabilitation a particularly attractive means to improve physical function and promote exercise. First, given the required immunosuppressive regimen, lung transplant recipients are at high risk for respiratory infections. As the lungs are the only transplanted solid organ that is directly exposed to the environment, high risk for respiratory infection poses a concern when leaving home to attend group-based rehabilitation programs. Second, the post-transplant regimen involves frequent medical appointments and complications that may necessitate brief hospital readmissions and interruptions in structured programs. In fact, the highest rate of hospital readmission occurs within the first 30 to 90 days following lung transplantation (Chan et al., 2016), a critical period when lung transplant recipients are actively participating in rehabilitation programs. Telerehabilitation offers the potential of providing sessions in a more flexible manner to deal with unexpected schedule changes. Third, the ability to exercise at home with intermittent, rather than direct, clinician supervision may be a critical factor in establishing behaviors that facilitate long-term adherence to exercise self-management. The optimal exercise program for this population is likely one that is flexible, convenient, in-place, and promotes self-management of exercise. Telerehabilitation seems ideally suited to meet the needs of this unique population.

We therefore developed and pilot-tested a telerehabilitation-based exercise program as a sustainable means to overcome the unique barriers to exercise faced by patients following lung transplantation. The goals for this pilot study were to: (1) evaluate the feasibility, safety, system usability, and intervention acceptability of *Lung Transplant Go (LTGO),* an 8-week, in-home exercise intervention for lung transplant recipients using a telerehabilitation platform; and (2) describe changes in physical function and physical activity from baseline to one week after completion of the 8-week intervention.

## METHODS

### DESIGN, SETTING AND SAMPLE

We used a single group, within-subjects pre- and post-test design. Our study protocol was approved by the University of Pittsburgh Institutional Review Board. Participants were recruited from the University of Pittsburgh Medical Center (UPMC) Lung Transplant program. Clinical team members in the UPMC Lung Transplant program identified eligible participants and requested their permission to be contacted by a research team member. When granted, a research team member contacted potential participants, verified eligibility, introduced the study, and obtained informed consent.

Eligibility criteria were: (1) age ≥ 18 years old; (2) received a lung transplant within the past 3 months and currently discharged from the hospital; (3) physician approval for participation; (4) reliable phone access; (5) able to speak and read English. Exclusion criteria were: (1) currently enrolled or planned to enroll in a formal pulmonary rehabilitation program (dual enrollment would confound ability to measure program outcomes); and (2) residing at a distance that would require more than a 3-hour drive from the UPMC Lung Transplant program (home visits required for system set-up).

### TECHNOLOGICAL STRUCTURE FOR THE TELEREHABILITATION PLATFORM

**Figure 1:**
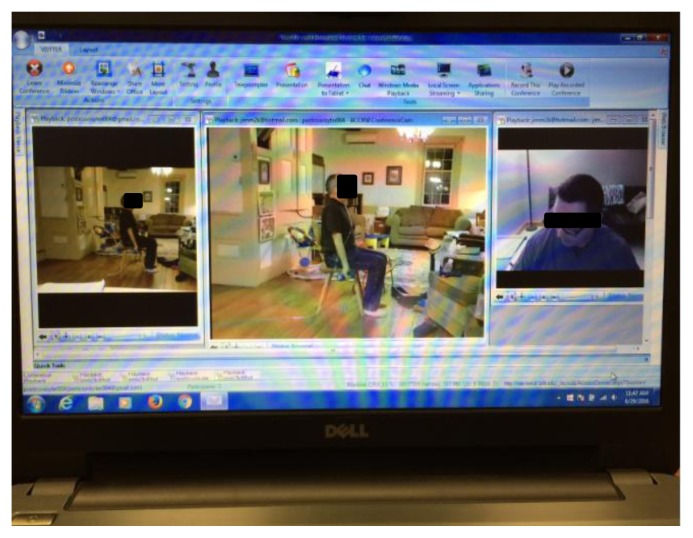
A sample screenshot of an exercise session delivered via VISYTER.

The telerehabilitation platform, Versatile and Integrated System for TeleRehabilitation (VISYTER) was selected due to its interactive capabilities, simplicity for implementation in the home setting, and high acuity of video imaging (Parmanto et al., 2010). The main capabilities of VISYTER used for the LTGO intervention include (1) video conferencing, (2) camera control that allowed the interventionist to remotely change the visual field using the pan-tilt-zoom mode, (3) remote real-time demonstration of exercise and observation of responses, and (4) eye contact and a teleprompter, an essential feature to help participants perceive credibility and promote flow of communication. Participants were assigned an individual user ID and password to log into the system and individual virtual clinic room. Features in the VISYTER system were designed to assure privacy, security, and confidentiality requirements. All data were encrypted. All virtual clinic rooms were housed on a server which restricted accessibility based upon the user’s role in the study. For the videoconferencing system, two web cameras were used (Logitech HD C910 and Logitech BCC 950) for both the participant and the interventionist. One of the cameras, Logitech BCC 950**,** had zoom and tilt functions and a speaker that allowed the interventionist to control angles and zoom remotely. Web cameras were connected to a 15.6 inch laptop computer (Dell Inspiron 15R PC with Windows® 7). Video and audio data were encrypted and transmitted over a high-speed Internet connection requiring as low as 384 kbps for both upload and download capacity. Both the interventionist and participant had the same system. A screenshot of the VISYTER system is depicted in Figure 1. All sessions were videotaped to facilitate evaluation of delivery approaches, participant response, and fidelity of the intervention.

### LTGO INTERVENTION

The intervention was an 8-week in-home exercise program that focused on instruction in an individualized aerobic and strengthening exercise program. The program was developed in consultation with a physical therapist and an exercise physiologist, and was delivered on a weekly basis to each participant. Exercises included warm-up and cool down exercises, strengthening exercises (using cuff weights), aerobic exercise (walking) and balance exercises to promote endurance, flexibility, balance, and strengthening. Exercise prescription and progression were based on guidelines of the American College of Sports Medicine (ACSM) and American Association of Cardiovascular and Pulmonary Rehabilitation (Collins et al., 2014). During the initial home visit, the interventionist assessed participants’ physical function, discussed goals and preferences, developed an exercise regimen, and instructed participants regarding how to carry out the exercises. The regimen was advanced, maintained, or reduced based on review by the interventionist during weekly LTGO sessions. When exercising independently, participants were instructed to stop exercise if (1) arterial oxygen saturation measured by pulse oximetry (SpO_2_) decreased below 90%, (2) heart rate increased above 130 beats per minute, (3) the Modified Borg dyspnea scale (0–10) was rated greater than 4 (somewhat severe), or (4) the participant experienced any signs of discomfort or distress. If symptoms did not resolve within 15 minutes, participants were instructed to inform the interventionist who was then expected to re-evaluate the exercise regimen and seek medical advice, if necessary. Details of the intervention protocol are summarized in Table 1.

### DATA COLLECTION AND MEASURES

At baseline (pre-intervention) and after completion of the 8-week intervention (post-intervention), participants completed the following assessments of physical function: 6-minute walk distance (6MWD), Berg balance scale, and 30-second chair stand test. To insure consistency in measurement, data were obtained by a licensed physical therapist at the University of Pittsburgh Physical Therapy Clinical and Translational Research Center (PT-CTRC). To evaluate physical activity, participants were asked to wear a SenseWear Armband® for 7 days to prior to and after completing the 8-week intervention. Data recorded by the device were downloaded and analyzed using manufacturer software. At post-intervention, participants completed a questionnaire to assess usability of VISYTER in delivering the home exercise program (Telehealth Usability Questionnaire) and participated in a semi-structured interview to determine intervention acceptability. These measures were obtained by a research team member in a private conference room located in the PT-CTRC.

### STUDY VARIABLES AND MEASURES

#### PHYSICAL FUNCTION

The *6-Minute Walk Test (6MWT)* was used to measure exercise capacity (American Thoracic Society, 2002). The 6MWT is a standardized, well-validated measure of exercise capacity in people living with chronic respiratory conditions. The 6MWT was performed in a 37.56 meter indoor track in the PT-CTRC. Participants were asked to walk as far as possible in 6 minutes. Each minute, SpO_2_, heart rate and the Borg scale (6–20) of perceived exertion (breathing, leg fatigue) were measured. Participants were allowed to rest if they felt necessary. Participants who required oxygen during exercise used supplemental oxygen at the prescribed flow rate during the test.

*30-second Chair-Stand Test* was used to measure lower body strength (Jones, Rikli, & Beam, 1999). The test measures the number of chair stands completed by the participant in 30 seconds. For chair stands, participants were asked to start from the seated position and repeat standing up and sitting down on the chair with both arms crossed against the chest. This test has been validated for measurement of lower body strength in older adults with COPD (Benton & Alexander, 2009).

*Berg Balance Scale* was used to measure balance (Berg, Wood-Dauphinee, & Williams, 1995; Berg, Wood-Dauphinee, Williams, & Maki, 1992). The Berg Balance Scale consists of 14 tasks including sitting to standing, standing unsupported, and picking up object from the floor from a standing position. Each task is scored on a scale of 0 (unable to perform a task) to 4 (able to perform the task independently). Possible scores range from 0 to 56 with higher scores indicative of better performance on the measure.

#### PHYSICAL ACTIVITY

*SenseWear Armband®* (Body Media, Pittsburgh, PA) was used to measure 7-day physical activity. The armband sensors detect movement, and data from this measure was used to determine the average number of steps walked daily. Acceptable validity when compared against indirect calorimetry (Cereda et al., 2007; Fruin & Rankin, 2004; Jakicic et al., 2004) and other activity monitors (e.g., accelerometers) has been reported (King, Torres, Potter, Brooks, & Coleman, 2004). Participants were asked to wear the armband for seven days (except when bathing) at baseline (pre-intervention) and post-intervention.

#### FEASIBILITY

Assessed by recording the number of interventionist supervised exercise sessions completed and time (weeks) required for completion.

#### SAFETY

Assessed by recording the number and type of adverse events and any remedial action recorded in the patient’s exercise diary and interventionist records.

#### SYSTEM USABILITY

*Telehealth Usability Questionnaire* (Faett, Brienza, Geyer, & Hoffman, 2013) was completed post intervention to assess usability of the VISYTER in delivering the home exercise program. This 21-item measure contains items that assess 6 subscales of usability: usefulness, ease of use, interface quality, interaction quality, reliability and satisfaction. Participants rated each item using a Likert scale (1=disagree to 7=agree). Scores on individual items are summed to obtain a mean total score (range 1–7). Higher scores indicate greater usability.

#### INTERVENTION ACCEPTABILITY

A semi-structured interview was conducted with participants by telephone or in-person following completion of the intervention. Participants were asked to describe their: reason for study participation, whether the overall experience was helpful or not-helpful, suggestions for improvement, and willingness to participate in a similar intervention if available. Each interview lasted 7–23 (median 12.5) minutes.

## RESULTS

### SAMPLE CHARACTERISTICS

Demographic and clinical characteristics of the four participants are summarized in Table 2. Participants’ age ranged from 30 to 66 years. All were Caucasian; three were male, and three had a pretransplant diagnosis of idiopathic pulmonary fibrosis. Three received a double and one a single lung transplant. One participant (#3) required supplementary oxygen during exercise pre- and post- intervention. All participants stated they were familiar with information technology as they used a computer or smart phone for email, shopping, calendar entries, travel arrangements and social networking.

### PHYSICAL FUNCTION AND PHYSICAL ACTIVITY

**Figure 2:**
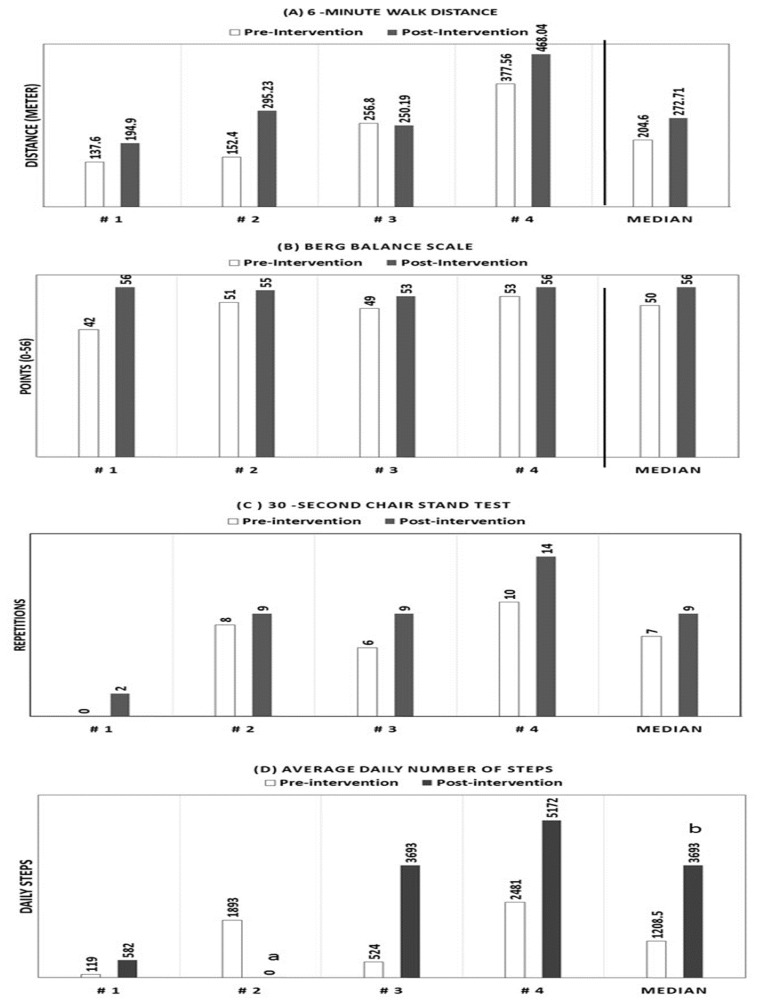
Measures of physical function and physical activity pre- and post- intervention (N=4) (B) Berg Balance Scale, Possible score range 0–56. Higher score indicate better balance. ^a^ Missing data (Participant did not wear the armband); ^b^ n=3 due to missing data

Results for measures of physical function are presented in Figure 2. The 6MWD improved in 3 of the 4 participants with a median increase of 90 meters. All participants improved scores on the Berg Balance Scale (median increase 4 points) and the 30-second chair stand test (median increase 2.5). At baseline, participants walked a median of 1209 daily steps (range 119–2481 steps). After completion of the intervention, 3 participants provided activity readings. These participants walked a median of 3693 daily steps (range 582–5172 steps).

### FEASIBILITY AND SAFETY

Three participants completed eight sessions and one (# 2) completed 7 of 8 sessions. Each session took an average of 42 minutes (range 15 – 75 minutes). Completion required an average of 10 weeks (range 8 – 13 weeks). Reasons for cancellation or delay were transplant-related complications (e.g., infection, acute rejection or hospital readmission) which temporarily led to postponement of the scheduled exercise session. One participant was readmitted to the hospital during the intervention period. This participant was able to resume the exercise program after hospital discharge. No adverse events were recorded.

### SYSTEM USABILITY

Participants rated usability of the VISYTER system as high. The median score for the Telehealth Usability Questionnaire was 6.05 (range 5.76 – 6.85). No major technical problems were reported. Median scores for the 6 usability sub-scales are summarized in Table 3.

### INTERVENTION ACCEPTABILITY

Main points and sample quotes relating to acceptability are summarized in Table 4. When asked why they chose to participate in the intervention, all participants reported that they believed that exercise was essential to improve their strength and balance. Participants hoped to become disciplined in doing regular exercise and viewed the intervention as promoting accountability. Participants also reported that the absence of need for additional travel was a desired aspect of the program.

All participants reported a positive experience with the telerehabilitation program, noting that the intervention promoted improved physical function (strength, balance, aerobic capacity), provided realistic goal setting and pacing, and allowed flexibility in scheduling. Participants enjoyed the interactive nature of the intervention and the format which made it easy to get back on track if exercise was interrupted due to illness. They reported minimal difficulty using the technology; problems related to the technology were able to be resolved during the first session. Several reported an occasion when network connectivity was lost, but also commented that while this was briefly problematic, the issue was resolved without affecting the flow of the intervention.

When asked if they would participate in a similar exercise program, all responded positively. The ability to avoid exposure to infection from contact with other patients in a group setting was particularly important, as noted by one participant:

“When you are in your home and doing something like this, the environment is safe. If you go to a clinic, (although) I know they are keeping things clean and doing what they can, but there are still people. They might not now know that they are sick but it is coming on. I have gotten sick a couple times and it does not go away quick. A little cold affects me for days or weeks. That part is very important. Even if I lived right next to a pulmonary rehab center, there would be times where I would much rather do a videoconference in my own home.”

When asked about ways to improve the intervention, participants suggested including more support to help them develop behavioral strategies to incorporate their needs of physical exercise into their daily life.

## DISCUSSION

To our knowledge, this is the first study to test a home exercise program delivered via a telerehabilitation system to lung transplant recipients. Our results support the feasibility, safety, usability, and acceptability of this intervention and improvement in physical function and physical activity in four participants.

Our participants were able to show improvement in physical function, including 6MWD, balance, and lower body strength. Of importance, improvement in 6MWD for three of four participants exceeded the minimal clinically importance difference (54 meters) (Redelmeier, Bayoumi, Goldstein, & Guyatt, 1997). One participant showed a slight decrease in 6MWD, but had improvements in both balance and lower body strength. This individual was the only single lung transplant recipient and remained oxygen dependent following transplant with marginal pulmonary function, factors that may have explained the decrease in 6MWD.

When pre and post intervention measures were compared, all participants showed improvement in physical activity, measured by average daily steps walked. This finding supports an important benefit because improvement in measures of physical function has not been shown to consistently translate into increased physical activity (Singh, 2016). While promising, with exception of the youngest participant (#4), the extent of improvement did not reach the number of daily steps recommended for populations living with chronic health conditions (3500–5000 steps per day) (Tudor-Locke & Myers, 2001). Our intervention was limited to 8 weeks and post-intervention measurement occurred immediately following completion of the intervention period. Given the early stage of recovery (all enrolled within three months post-transplant), further time may be required to reach this goal. Notably, our intervention did not include behavioral strategies designed to promote exercise adherence. To accomplish this goal, it may be important to incorporate behavioral strategies in future studies to promote physical activity in lifestyle and extend follow-up for a longer interval.

From participant interviews, we were able to identify important benefits unique to exercise delivery via telerehabilitation. All participants commented that the experience of “building exercise routines” in their home was the most attractive aspect of training via the VISYTER system. Prior studies consistently report difficulty maintaining benefits of exercise training. Telerehabilitation provides the ability to initiate and supervise exercise in a familiar setting, a benefit that may promote adherence to the regimen over time. Participants valued flexibility in scheduling and pacing, an accommodation critical to lung transplant recipients who may face challenges such as multiple doctor appointments, illness due to transplant related complications, and hospitalization. With the VISYTER system, sessions could be scheduled over a variable range of time (range 8–13 weeks). Such flexible scheduling would not be possible if enrolled in a structured pulmonary rehabilitation program.

Walsh et al. (2013) evaluated recovery in exercise capacity, quadriceps muscle strength, and lung function over the first 26 weeks after transplant and found there was dyssynchronous improvement in these measures. Rather than being predicted by improved graft function, improved exercise capacity was explained by pre-transplant exercise capacity and post-transplant improvement in quadriceps muscle strength (Walsh et al., 2013). Our intervention allows variation in the regimen that matches individual functional ability and therefore may be more effective in improving muscle strength. Langer et al (2012) compared outcomes in lung transplant recipients randomized to 3 months of supervised training immediately after hospital discharge versus usual care. They reported significant improvements in quadriceps force, 6MWD, and walking time in the intervention group at 12 months. However, almost 40% of eligible candidates refused to participate in the trial, which required participation in supervised exercise 3 times weekly for 3 months at a clinic facility (Langer et al., 2012). The authors speculated that long travel distances might have been a factor that discouraged participation (Langer et al., 2012). Our intervention, because it is provided in the home setting, would eliminate this disadvantage.

Our results are consistent with prior studies evaluating use of VISYTER in individuals living with other complex medical conditions. VISYTER was evaluated positively when used to provide remote wheelchair assessment (Schein, Schmeler, Brienza, Saptono, & Parmanto, 2008; Schein, Schmeler, Saptono, & Brienza, 2010), to provide home instruction in use of leg compression devices to reduce lymphedema in adults with limited mobility (Faett et al., 2013; Faett, Geyer, Hoffman, & Brienza, 2012), and to diagnose autism in adults through remote evaluation of facial expression, language and behaviors (Parmanto et al., 2013). These studies illustrate the diversity of potential uses of this system.

Our study has several limitations, including small sample size, absence of a control group and limited diversity with regard to gender and the underlying diagnosis resulting in need for transplant. Thus, the ability to generalize our results is limited. Due to the pilot nature of our study, we limited duration of the intervention to 8 weeks and carried out post testing immediately following completion of the intervention. Therefore, we were unable to evaluate ability of the intervention to produce long-term impact in physical function and physical activity.

## CONCLUSIONS

Our findings support the feasibility of recruitment and retention of lung transplant recipients during the early post-transplant period, safety of delivering an exercise intervention via a telerehabilitation platform, and acceptability of the approach. Notably, our findings provide preliminary evidence for the ability of the LTGO exercise intervention to improve physical function in important ways that include walk distance, balance, and lower body strength in lung transplant recipients. Future studies are needed to determine if the benefits shown in this study can be enhanced by the addition of behavioral strategies to promote self-management of exercise in a larger sample, followed over a longer period of time.

## Figures and Tables

**Table 1:** Intervention Protocol

**Step 1. System set-up and face-to-face instruction** **System set-up:** The interventionist sets up the VISYTER system in the participant’s home and provides instruction regarding its use. Access to a high speed Internet is provided if participants’ current access is not fast enough. **Planning:** The interventionist (1) reviews the participant’s baseline physical function and clinical data, (2) discusses participant’s goals and preference and (3) develops a daily exercise regimen. **Instruction:** Participants receive instructions for (1) the daily exercise regimen, (2) keeping an exercise diary, and (3) using monitoring devices (i.e., a pulse oximeter, automatic blood pressure monitor, and a pedometer). Participants also receive a 5–10 lbs. cuff weight (adjustable by 0.5 lbs.) and exercise instruction booklet with illustrations and text.
**Step 2. 8 weeks of exercise** Weekly LTGO sessions (≈ 40 minutes each x 8 sessions): An interventionist and the participant log in to the VISYTER system weekly. During each session, the interventionist (1) assesses type, frequency and duration of concurrent usual care and health care service use (e.g., emergency visits, hospitalization); (2) reviews and discusses the participant’s exercise diary from the previous week; (3) asks the participant to demonstrate exercises to evaluate the current exercise regimen; and (4) teaches exercises he/she needs to do for the next week after determining if the regimen will be maintained, advanced, or reduced. Further instruction and demonstration are given as necessary. **Note:** All sessions are recorded via the VISYTER video archiving function to monitor intervention fidelity and identify facilitators and barriers. **Daily self-exercise for 8 weeks:** Participants are instructed to practice their exercises daily and keep an exercise diary, monitor SpO2 and blood pressure, and record daily steps (with a pedometer). The exercise booklet is also used to supplement instruction as needed.
**Step 3. Intervention wrap-up** **System extraction:** Participants return the VISYTER system, pulse oximeter, pedometer, automatic blood pressure monitor, and completed exercise diary when they visit the PT-CTRC for post-intervention evaluation. **Debriefing interview:** The PI conducts a semi-structured interview to evaluate the participants’ experience in the 8-week LT-VISYTER program.

**Table 2:** Baseline Sample Characteristics (N=4)

Participant ID #	1	2	3	4
Gender	Male	Male	Male	Female
Age, years	66	62	62	30
Ethnicity	Caucasian	Caucasian	Caucasian	Caucasian
Pre-Transplant diagnosis	IPF	IPF	IPF	CF
Type of Transplant	Double	Double	Single	Double
Time between transplant and study enrollment days	77	33	75	36
Hospital LOS, days	71	9	28	18
Body Mass Index (kg/m^2^)	27.7	22.9	28.0	15.6
FEV_1_ Actual (L)/ % predicted	1.90/ 58	3.89/ 109	1.91/ 61	1.52/ 57
FVC Actual (L)/ % predicted	2.01/ 42	4.64/ 90	2.27/ 51	1.65/ 50

LOS = Length of Stay; IPF = Idiopathic Pulmonary Fibrosis; CF = Cystic Fibrosis; BMI, FEV1 and FVC are the values obtained at study baseline.

**Table 3:** Subscale Scores of the Telehealth Usability Questionnaire (N=4)

Subscales	Median (Range)
Usefulness	6.50 (5.67 – 7.00)
Ease of use, learnability	6.33 (5.67 – 7.00)
Interface quality	6.25 (5.25 – 7.00)
Interaction quality	6.57 (5.29 – 6.86)
Reliability	6.75 (6.00 – 7.00)
Satisfaction and future use	7.00 (7.00 – 7.00)

Likert scale (1=Disagree; 7=Agree). Score range 1–7. Higher score indicates better usability.

**Table 4:** Main Highlights and Sample Quotations from Acceptability Interview

Experience with the intervention
Improved physical function *“It helped me to stay on track and to do regular exercises. It helped with my aerobics, muscles and stretching. I felt that when I was doing it with that schedule it was good – and I felt better.”* (Quotation 1)
Realistic goal setting and pacing, and flexibility *“The trainer knew what we could do and what we couldn’t do. As we progressed, he was able to progress with me. He would do more with me- add more weight, add more time. Without him, it wouldn’t have worked well…. Because you aren’t physically in very good shape- you really need to start slowly and build yourself up. If you started right in with then it just wouldn’t work because you just can’t physically do it and you would get discouraged.”* (Quotation 2)
Interactive nature of the intervention *“The interaction was very good when we were together on camera. Rather than just having a videotaped program.”* (Quotation 3)
Helpful in getting back on track *“Another thing that happened for me, when I get sick, which I’ve been back and forth, then it kind of breaks the cycle of doing the exercises. But it’s been good though. Also, I use the same plan when I do my exercise and I add some things to it but you have to have the outline first.”* (Quotation 4)
Simple technology *“Basically, the one problem was with the one camera. It didn’t always work for the trainer. But if I played around with the up and down button then he didn’t have any problem. Other than that technical problem – which was easy to fix – he didn’t have anything wrong there. He could see if I was lifting my legs correctly or properly doing the weights. I think the technology in general was good.”* (Quotation 5)
Thoughts and suggestions on future interventions
Willingness to participate in a similar exercise intervention. *“Yes I would. I need the discipline after the transplant. The trouble with a transplant is… all of a sudden you start to feel so well, and then you take things for granted and you back slide. I feel that I don’t have enough self-control or discipline. I was sorry to see the program end. I really felt that I should have done more. That’s the trouble with lung transplant patients. You start to feel good, you go back to work and then you stop doing your exercises. I just don’t know how if work 12 hours a day now and now I’m too tired to exercise but if you have someone tell you that you’re going to do it then you do.”* (Quotation 6) *“Anything that would motivate me to get off the couch and get out of bed. I think anything that I feel responsible – it’s like this call that is really important to me. We make a plan to be on the phone at this time- it’s really important to me.”* (Quotation 7)
Incorporating behavioral strategy will be beneficial. *“Maybe they could ask, ‘How did it go? How did you do it?’ For example, if it raining or snowing out they could ask me, ‘what did you do?’ and I would probably respond I got off of my couch and moved over a couple of times (laughs) that was my excuse, or I don’t have any snow shoes. Well, I do have a bike and I could pump away on my back. But I’m not a very physical person- I believe exercise is life is that you need to be busy and not on a couch. Anyway, for me it would have encouraged me to get me off my butt.”* (Quotation 8)
